# Comparison of sequential multiplex PCR, sequetyping and whole genome sequencing for serotyping of *Streptococcus pneumoniae*

**DOI:** 10.1371/journal.pone.0189163

**Published:** 2017-12-13

**Authors:** Florian Mauffrey, Éric Fournier, Walter Demczuk, Irene Martin, Michael Mulvey, Christine Martineau, Simon Lévesque, Sadjia Bekal, Marc-Christian Domingo, Florence Doualla-Bell, Jean Longtin, Brigitte Lefebvre

**Affiliations:** 1 Laboratoire de santé publique du Québec, Institut national de santé publique du Québec, Sainte-Anne-de-Bellevue, Québec, Canada; 2 National Microbiology Laboratory, Public Health Agency of Canada, Winnipeg, Manitoba, Canada; Universidade de Lisboa Faculdade de Medicina, PORTUGAL

## Abstract

*Streptococcus pneumoniae* is one of the major causes of pneumonia, meningitis and other pneumococcal infections in young children and elders. Determination of circulating *S*. *pneumoniae* serotypes is an essential service by public health laboratories for the monitoring of putative serotype replacement following the introduction of pneumococcal conjugate vaccines (PCVs) and of the efficacy of the immunization program. The Quellung method remains the gold standard for typing *S*. *pneumoniae*. Although this method is very effective, it is also costly, time consuming and not totally reliable due to its subjective nature. The objectives of this study were to test and evaluate the efficiency of 3 different molecular methods compared to the Quellung method. Sequential multiplex PCR, sequetyping and whole genome sequencing (WGS) were chosen and tested using a set of diverse *S*. *pneumoniae*. One-hundred and eighteen isolates covering 83 serotypes were subjected to multiplex PCR and sequetyping while 88 isolates covering 53 serotypes were subjected to WGS. Sequential multiplex PCR allowed the identification of a significant proportion (49%) of serotypes at the serogroup or subset level but only 27% were identified at the serotype level. Using WGS, 55% to 60% of isolates were identified at the serotype level depending on the analysis strategy used. Finally, sequetyping demonstrated the lowest performance, with 17% of misidentified serotypes. The use of Jin *cpsB* database instead of the GenBank database slightly improved results but did not significantly impact the efficiency of sequetyping. Although none of these molecular methods may currently replace the Quellung method, WGS remains the most promising molecular pneumococcal serotyping method.

## Introduction

The Gram-positive lancet-shaped cocci bacteria *Streptococcus pneumoniae* is frequently associated with meningitis, pneumonia and sepsis in humans in addition to be the major cause of mortality in children [1]. Pneumococcus infections mainly occur among young children and the elderly, under 5 years old and above 65 years old, respectively [2]. More than 90 *S*. *pneumoniae* capsular polysaccharide (CPS) types exist resulting in a large variety of serotypes belonging to 46 different serogroups [3]. In Canada, the introduction of the seven-valent pneumococcal conjugate vaccine (PCV-7) in 2005 targeting the seven predominant serotypes (4, 6B, 9V, 14, 18C, 19F, and 23F) led to a significant decrease in invasive pneumococcal diseases (IPD) associated to these serotypes [4]. However, replacement of vaccine serotypes by non-vaccine serotypes (NVT) led to the emergence of serotype 19A as the new predominant multi-drug resistant serotype [5]. Following the advent of NVT, two others vaccines were released in 2008 and 2010, PCV-10 and PCV-13, respectively. The monitoring of IPD serotypes became essential as new NVT may have emerged making the introduction of new vaccines necessary.

Serotyping methods of *S*. *pneumoniae* can be grouped in two different categories: phenotype-based methods and genotype-based methods [6]. The Quellung method (based on antisera reactions) still remains the Gold Standard method used in most laboratories [7]. However this method is expensive, laborious and not fully reliable. Following the sequencing of the *cps* loci of 90 pneumococcal serotypes, methods based on the detection of serotype-specific genes were developed in order to provide cost-effective and reliable assays for the serotyping of *S*. *pneumoniae* [6,8].

Among these methods, three were chosen for comparison in this study: sequential multiplex PCR, sequetyping and whole genome sequencing (WGS). The sequential multiplex PCR protocol developed by the Centers for Disease Control and Prevention (CDC) relies on the use of primers targeting serotype- or serogroup-specific regions in the *cps* loci [9]. PCR has been extensively used for the serotyping of *S*. *pneumoniae* and had the advantage of being easy to use and can be performed on a large quantity of samples [10–13]. The sequetyping method was developed by Leung *et al*. (2012) and is based on the *cpsB* gene sequence which appears to be specific to serotypes [14]. WGS became a suitable method for serotyping with the improvement in accuracy and a decrease in cost which has allowed the identification of serotype by comparing *cps* loci sequences [15–17].

The replacement of the Gold Standard Quellung method in routine laboratories by a genotype-based method is a current issue for many laboratories, requiring preliminary estimations of the efficiency and adaptability of different methods. Such comparisons and evaluations for some methods have already been conducted [18–22]. Unfortunately, inter-strain genome variations led to an increase in *cps* loci rearrangement and diversity. Thus the efficiency of molecular serotyping methods may vary between strains and/or between different regions [8,23].

In this study, a large number of serotypes were included, but a focus on the most prevalent serotypes in Québec/Canada and serotypes targeted by PCV-13 were chosen. The evaluation of a potential molecular replacement for the Quellung identification method was considered.

## Material and methods

### Isolates, culture conditions and DNA extraction

**Fig 1:**
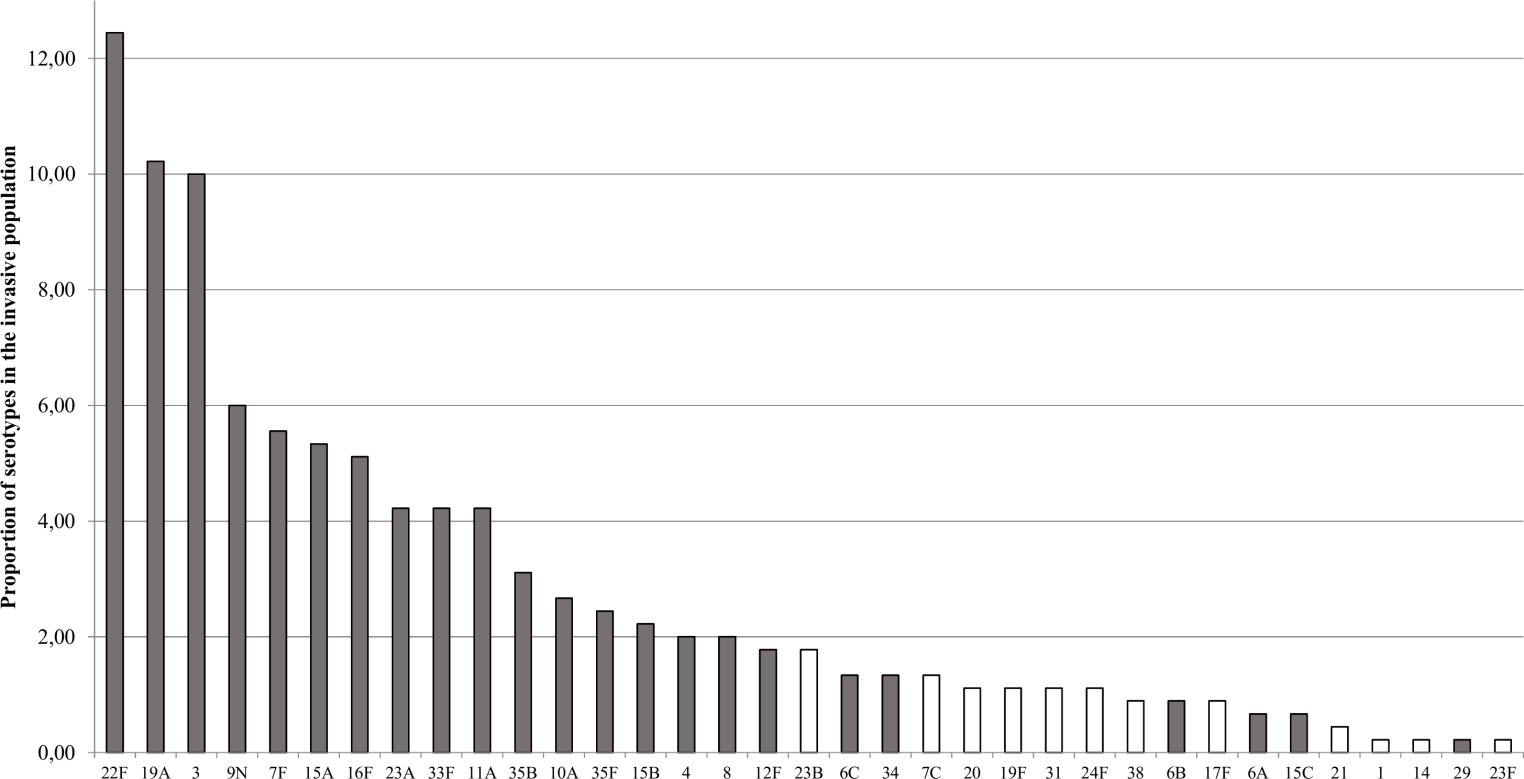
S. pneumoniae serotype distribution. Serotype distribution in the province of Québec in 2016. Grey bars represent serotypes tested by WGS in this study.

One hundred eighteen invasive *S*. *pneumoniae* representing 83 serotypes previously identified by the Quellung reaction were selected from the Laboratoire de santé publique du Québec (LSPQ) provincial surveillance program (see S1 Table). All the isolates were subjected to sequential multiplex PCR and sequetyping methods. Six serotype 35A isolates and six serotype 34 isolates were added to the pool tested with the sequential multiplex method as well as six serotype 29 isolates were added to the sequetyping pool. A subset of 53 isolates were tested with WGS and represented 32 different serotypes. The selection of the serotypes was performed on the basis of the most prevalent serotypes in the province of Québec in 2012–2016 (Fig 1). Rare serotypes were also included in order to test the robustness of the method. WGS data for 35 *S*. *pneumoniae* was also provided by the National Microbiology Laboratory (NML, Winnipeg, Canada), totaling 88 isolates representing 53 serotypes subjected to serotyping using WGS approach. Finally, three *Streptococcus pseudopneumoniae* and three *Streptococcus mitis* were used as specificity controls for sequential multiplex PCR and sequetyping.

Isolates were cultured on TSA II (Trypticase Soy Agar with 5% sheep blood) agar plate and incubated overnight at 35°C in a 5% CO_2_ atmosphere. Bacteria were collected with a loop and suspended in G2 buffer solution with RNase A (QIAGEN inc, Toronto, ON, Canada). Samples were then frozen at -20°C until extraction. DNA extraction was performed with the MagAttract DNA Mini M48 Kit (QIAGEN inc, Toronto, ON, Canada) and the QIAGEN^TM^ BioRobot M48 workstation according to manufacturer’s instructions.

### Sequential multiplex PCR

The CDC sequential multiplex PCR protocol was used as described by Carvalho *et al*. (2010). Briefly, primers pairs were designed to target serotype- or serogroup-specific regions in the *wzy* or *wzx* genes. The choice of primers was modeled on those included in the CDC protocol as they were adapted to the 22 most prevalent serotypes in Quebec (2012–2016). These serotypes represent 90% of IPD in Quebec. All serotypes included in the PCV-13 (4, 6B, 9V, 14, 18C, 19F, 23F, 1, 5, 7F, 3, 6A and 19A) were also covered by this protocol. Positive and negative controls were used in each reaction. Positive controls consisted of a mix of *S*. *pneumoniae* DNA extract of serotypes present in each multiplex. *S*. *pseudopneumoniae* and *S*. *mitis* DNA extracts were tested in each multiplex as a control of specificity.

### Sequetyping

Sequetyping procedures were conducted as described by Leung *et al*. (2012) with some modifications. Briefly, master mix was composed of 0.3 μl of Amplitaq DNA polymerase (5 U/μl), 38.85 μl of DNA-free water, 5 μl of 10x PCR buffer (ThermoFisher Scientific, Whitby, Canada), 1.5 μl of MgCl_2_ (50 mM), 0.75 μl of dNTPs (10 mM), 0.8 μl of *cps1* and *cps2* primers (25 μM) and 2 μl of DNA extract for a final volume of 50 μl. Cycling conditions was performed as described by Leung *et al*. (2012). Sequencing was performed using the BigDye® Terminator v3.1 Cycle Sequencing Kit (ThermoFisher Scientific, Whitby, Canada) in a 3130*xl* Genetic Analyzer (ThermoFisher Scientific, Whitby, Canada).

Assembled *cpsB* sequences were blasted (default parameters) against a local and comprehensive *cpsB* database developed by Jin *et al*. (2016) [24]. This database extended the previous database created by Leung *et al*. (2012) by covering 95 serotypes instead of 93 and including a total of 390 sequences. Then, *cpsB* sequences were used to interrogate the GenBank database (https://www.ncbi.nlm.nih.gov/genbank/). In-house Python scripts allowed the automation of these processes. Hits with the highest identity value and High Scoring Pair (HSP) length were retained for serotype attribution. *cpsB* sequences were deposited in the GenBank database and are accessible under accession numbers MF693230-MF693347. Scripts have been deposited in the GitHub database and are available (https://github.com/Mauffrey/serotyping).

### Whole genome sequencing

Libraries for whole genome sequencing were prepared with the Nextera XT DNA library preparation kit and sequenced using an Illumina MiSeq reagent kit v3 (600 cycles, paired ends) following the manufacturer’s instructions. Reads quality was evaluated with FastQC (http://www.bioinformatics.babraham.ac.uk/projects/fastqc/). *De novo* genome assemblies were performed using SPAdes version 3.9.0 [25] assembler on Calcul Quebec public resources (http://www.calculquebec.ca/en/) with standard parameters and the option flag ‘—careful’ activated. Assemblies’ quality was assessed with Quast. Following assembling, in-house Python scripts allowed to remove contigs with length below 500 bp and coverage below 5x and to compute assembly’s statistics. Concerning the identification of the different *cps* loci, a local *cps* database was created with 107 *cps* sequences representing 92 different serotypes [3] retrieved from the NCBI GenBank database. Assembled contigs containing *cps* sequences were blasted against this database using BLAST+ tools suite in an automated in-house Python scripts. Serotypes were attributed considering hits with the highest bit scores. When multiple hits had high bit score and close identity values (<0.3% compared to best hit) for an equivalent HSP length, they were all retained for serotype attribution. FASTQ reads were deposited in the NCBI Sequences Reads Archive and are accessible under accession numbers SRR5962910-SRR5962997 (Bioproject accession number PRJNA398497). Scripts have been deposited in the GitHub database and are available (git@github.com:Mauffrey/serotyping.git).

PneumoCaT (Pneumococcus Capsular typing Tool), a serotyping designed workflow, was also used for serotype identification [26]. Automation of PneumoCaT was performed using a shell command based script. PneumoCaT uses a two-step pipeline for serotype attribution. The first step aligns raw reads with a stored *cps* sequence library to determine potential serotypes for an isolate. In a second step variant analysis is called only if several serotypes belonging to the same genogroup are returned from step 1 [26]. Briefly, reads are mapped against specific genogroup databases and functions are called for the detection of presence/absence of genes, allelic variants, single nucleotide polymorphisms (SNPs) and loss-of-function mutations. A serotype is returned at this point if a unique CPS locus is identified that is not a predefined genogroup, otherwise capsular loci variant analysis occurs for serotype attribution.

Isolates misidentified with the assembly-based strategy were subjected to further investigations. The *cps* locus was extracted from the corresponding contig according to the best hit coordinates and aligned with *cps* reference sequences of both best hit and expected serotype, for comparison. Alignments were done using the Artemis Comparison Tool (ACT) v6 and WebACT [27].

The impact of coverage and N50 on the serotyping quality between samples identified at the serotype level and samples identified as serogroup, subset or misidentified was evaluated with a Wilcoxon-Mann-Whitney test with an alpha value of 0.05 (see S5 Table).

### Serotype identification levels

For all the methods tested in this study, sample identification was classified as follows: 1) “Serotype” when a concordance was found with the Quellung and molecular identification methods, 2) “Serogroup” when a concordance was found with the Quellung identification as well as with other serotype(s) from the same serogroup, 3) “Subset” when a concordance was found with the Quellung identification as well as with other serotype(s) from a different serogroup, 4) “Misidentified” when no concordance was found with the Quellung and molecular identification methods and 5) “Not determined (N.D.)” when no amplification occurred in PCR multiplex reactions or when *cpsB* was not amplified in the sequetyping method. When isolates of the same serotypes had different identification levels with the same method, they were classified as inconsistent results when results per serotype were considered.

## Results

### Sequential multiplex PCR

Among all existing *S*. *pneumoniae* serotypes, the CDC sequential multiplex PCR protocol is able to detect 74 different serotypes. *cpsA* amplification ensures the presence of *S*. *pneumoniae* DNA in each reaction. In our experiments, *cpsA* amplification product was present in all reactions except for isolates of serotypes 25F and 38. The absence of amplification in those serotypes has been previously documented by Carvalho *et al*., (2010). Moreover, no *cpsA* amplification occurred with *S*. *pseudopneumoniae* and *S*. *mitis* isolates.

In this study, 130 isolates were tested with multiplex PCR method, covering 83 serotypes. Of the tested isolates, 45/130 (35%) were identified at the serotype level, 42/130 (32%) were identified at the serogroup level, 22/130 (17%) were identified at the subset level, 19/130 (15%) were not determined, and 2/130 (1%) were misidentified (Table 1A). All serotypes were not equally represented in our isolates selection, thus these results are not representative of the method efficiency concerning identification level. Nevertheless, all results were consistent when multiple isolates were tested for a same serotype, except for serotype 35A (1% of serotypes). Considering identification for each serotype, 22/83 (27%) were identified at the serotype level, 24/83 (29%) were identified at the serogroup level, 17/83 (20%) were identified at the subset level, 19/83 (23%) were not determined and 0/83 (0%) were misidentified (Table 1B). One serotype showed inconsistent results (serotype 35A), which accounted for 1% of the serotypes.

**Table 1:** Serotype identification results.

**A**	CDC sequential multiplex PCR (n = 130)	Sequetyping		WGS	
		NCBI online database (n = 124)	Curated *cpsB* database (n = 124)	Assembling strategy (n = 88)	PneumoCaT (n = 87)^(1)^
Serotype	35% (45)	49% (61)	52% (65)	60% (53)	70% (61)
Serogroup	32% (42)	16% (20)	16% (20)	28% (25)	0% (0)
Subset	17% (22)	11% (14)	10% (12)	7% (6)	0% (0)
Misidentified	1% (2)	19% (23)	17% (21)	5% (4)	30% (26)
N.D.	15% (19)	5% (6)	5% (6)	0% (0)	0% (0)
**B**	CDC sequential multiplex PCR (n = 83)	Sequetyping		WGS	
		NCBI online database (n = 83)	Curated *cpsB* database (n = 83)	Assembling strategy (n = 53)	PneumoCaT (n = 52)^(1)^
Serotype	27% (22)	42% (35)	46% (38)	55% (29)	59,5% (31)
Serogroup	29% (24)	14,5% (12)	17% (14)	24,5% (13)	0% (0)
Subset	20% (17)	14,5% (12)	14% (12)	11% (6)	0% (0)
Misidentified	0% (0)	17% (14)	16% (13)	5,5% (3)	36,5% (19)
Inconsistent	1% (1)	6% (5)	1% (1)	4% (2)	4% (2)
N.D.	23% (19)	6% (5)	6% (5)	0%	0% (0)

Results presented according to the 3 molecular methods tested and considering (A) isolates or (B) serotypes (number of isolates in brackets).

N.D. = not determinable (not detectable in CDC PCR protocol or *cpsB* not amplified).

^(1)^ One sample analysis failed because of too low reads number

Serotypes 34 and 35A showed unexpected results. Serotype 34 sample showed many amplicons, including a non-specific amplicon (250 bp) and the expected amplicon (408 bp), in the same reaction (multiplex PCR 7). Six more serotype 34 isolates were selected and subjected to identification with sequential multiplex PCR and the same non-specific amplification was present in 3 out of 6 reactions. The expected amplification product at 280 bp was not present in the multiplex PCR 7 with serotype 35A and 6 other serotype 35A isolates were further selected. For 5 out of 6 isolates, the expected amplicon was detected but a non-specific amplicon at 250 bp was also visible. It should be noted that expected amplicons bands are very well defined and have high intensity compared to non-specific amplicons bands which are generally less bright.

Non-specific amplification products were present in many PCR reactions. They seemed to occur randomly and did not depend on the isolate serotype. Only 4 different sizes non-specific amplicons were observed during this study, a non-specific bands at 500 bp in multiplex PCR 2, a non-specific band at 677 bp in multiplex PCR 3, a non-specific band at 850 bp in multiplex PCR 6 and a non-specific band at 250 bp in multiplex PCR 7. Except for the band at 677 bp in the multiplex PCR 3, these non-specific products did not correspond to expected product sizes in their respective multiplex PCR and were easily identified as non-specific. However, the amplification product at 677 bp in multiplex PCR 3 corresponds to the expected size for serotype 35B and is hardly identifiable as non-specific. Many non-specific amplicons were also present for *S*. *pseudopneumoniae* and *S*. *mitis* in most of the multiplex PCR.

### Sequetyping

Of the 124 *S*. *pneumoniae* isolates subjected to sequetyping, 118 (95%) were positive for *cpsB* amplification (1061 bp). No *cpsB* amplification was obtained for serotypes 25F, 37, 38, 39 and 43 which was in accordance with results from Leung *et al*. (2012) as these serotypes were predicted *in silico* to be non-amplifiable. However, no *cpsB* amplification was obtained with serotype 29 although it was expected to be amplifiable according to Leung *et al*. (2012). Therefore, 6 other serotype 29 isolates were selected and subjected to sequetyping. All 6 samples led to *cpsB* amplification. After sequencing and assembling, the average sequence length was 890 bp which is longer than the 732 bp region used by Leung *et al*., (2012) to test all their serotypes.

One hundred eighteen sequences representing 78 serotypes were subjected to BLAST for identification. Two different databases were chosen for the analysis: the exhaustive NCBI GenBank database and a more restrained but specific *cpsB* database created by Jin *et al*., (2016). Using the GenBank database, 61/124 (49%) were identified at the serotype level, 20/124 (16%) were identified at the serogroup level, 14/124 (11%) were identified at the subset level and 23/124 (19%) were misidentified. Using the Jin *cpsB* database, 65/124 (52%) were identified at the serotype level, 20/124 (16%) were identified at the serogroup level, 12/124 (10%) were identified at the subset level and 21/124 (17%) were misidentified (Table 1A). Inconsistent results were obtained for some serotypes (6B, 6C, 19F and 23F) when using the GenBank database but not using the Jin *cpsB* database. Considering only serotypes, identification with the GenBank database resulted in 35/83 (42%) identifications at the serotype level, 12/83 (14.5%) identifications at the serogroup level, 12/83 (14.5%) identifications at the subset level and 14/83 (17%) misidentified. With the Jin *cpsB* database, 38/83 (46%) were identified at the serotype level, 14/83 (17%) were identified at the serogroup level, 12/83 (14%) were identified at the subset level and 13/83 (16%) were misidentified. Results were slightly better with the Jin *cpsB* database (Table 1B), in particular for inconsistent results.

The majority of misidentifications were due to the attribution of closely related serotypes of the same genogroup [28]. For example, one serotype 9A isolate was identified as serotype 9V, one serotype 11F isolate was identified as serotype 11C and one serotype 42 isolate was identified as serotype 35B/35C see S2 Table in supplemental material for a complete and detailed list). For some misidentifications, there was no association between the determined serotype and the expected one. This was the case for one serotype 15C isolate identified as serotype 24F, one serotype 19F isolate identified as serotype 10A and one serotype 17A isolate was identified as serotype 10A. Serotype 29 isolates were all misidentified as serotype 35B/35C. Although these serotypes are genetically close, the percent similarity of our serotype 29 *cpsB* sequence compared with the serotype 29 reference sequence was only 83%.

Only one *S*. *pseudopneumoniae* isolate led to the amplification of *cpsB*. This sequence was associated with serotype 20 with 96% similarity which was the lowest score across all isolates.

### Whole genome sequencing

The number of paired-end reads obtained varied between 100 065 and 1 153 346 with an average of 542 388 (S3 Table). Assembling coverage varied from 14X to 296X and assembly’s length varied from 1 982 679 bp to 2 285 405 bp. Three strains presented unexpected features. LSPQ4282 and SC0268 assembly’s length were 6 793 942 bp and 2 698 601 bp, respectively, which is higher than the expected 2.0–2.2 Mbp length for *S*. *pneumoniae*. BLAST confirmed that *Bacillus subtilis* contamination occurred in LSPQ4282. After removal of *B*. *subtilis* associated contigs, assembly’s length dropped to 1 721 249 bp and coverage dropped from 36x to 6x. Assembly with and without *B*. *subtilis* sequences were both submitted to the serotyping pipeline and results were identical. SC0268 did not present any contamination with non-*Streptococcus* strains. However, it is possible that the sample consisted in a mix of several *S*. *pneumoniae* strains, leading to an assembly length longer than expected. MA080904 presented an unlikely mean coverage of 1012x. After investigation, it appeared that some small contigs had a very high coverage value, leading to a non representative mean coverage. After filtration of contigs with coverage > 1000x, a more representative coverage value of 37x was found.

Serotype identification was mainly based on sequence identity level and HSP length (S4 Table). For 53 of 88 isolates (60%), the serotype corresponded to that determined by Quellung without any ambiguity. The serogroup was determined for 25 of 88 isolates (28%), 6 of 88 (7%) were determined at the subset level and 4 of 88 isolates (5%) were misidentified. Considering serotypes, concordance with Quellung identification was found for 29 of 53 serotypes (55%), concordance at the serogroup level was found for 13 of 53 serotypes (24.5%), 6 of 53 (11%) were determined at the subset level and 3 of 51 (5.5%) were misidentified. Inconsistent results were obtained for isolates of serotype 6B and 7F, representing 4% of the serotypes tested. For some isolates, BLAST results could not discriminate between two different serotypes because of their high degree of genetic similarities or due to the existence of DNA polymorphism among single serotypes [29]. This was the case for 15B/15C, 22A/22F, 7A/7F, 11A/11D, 25A/25F, 32A/32F, 33A/33F, 9A/9V, 12A/46, 12F/44, 18B/18C and 35A/35C/42. Statistical analysis showed that coverage and N50 seemed to have no significant impact on the quality of the serotyping results.

**Fig 2:**
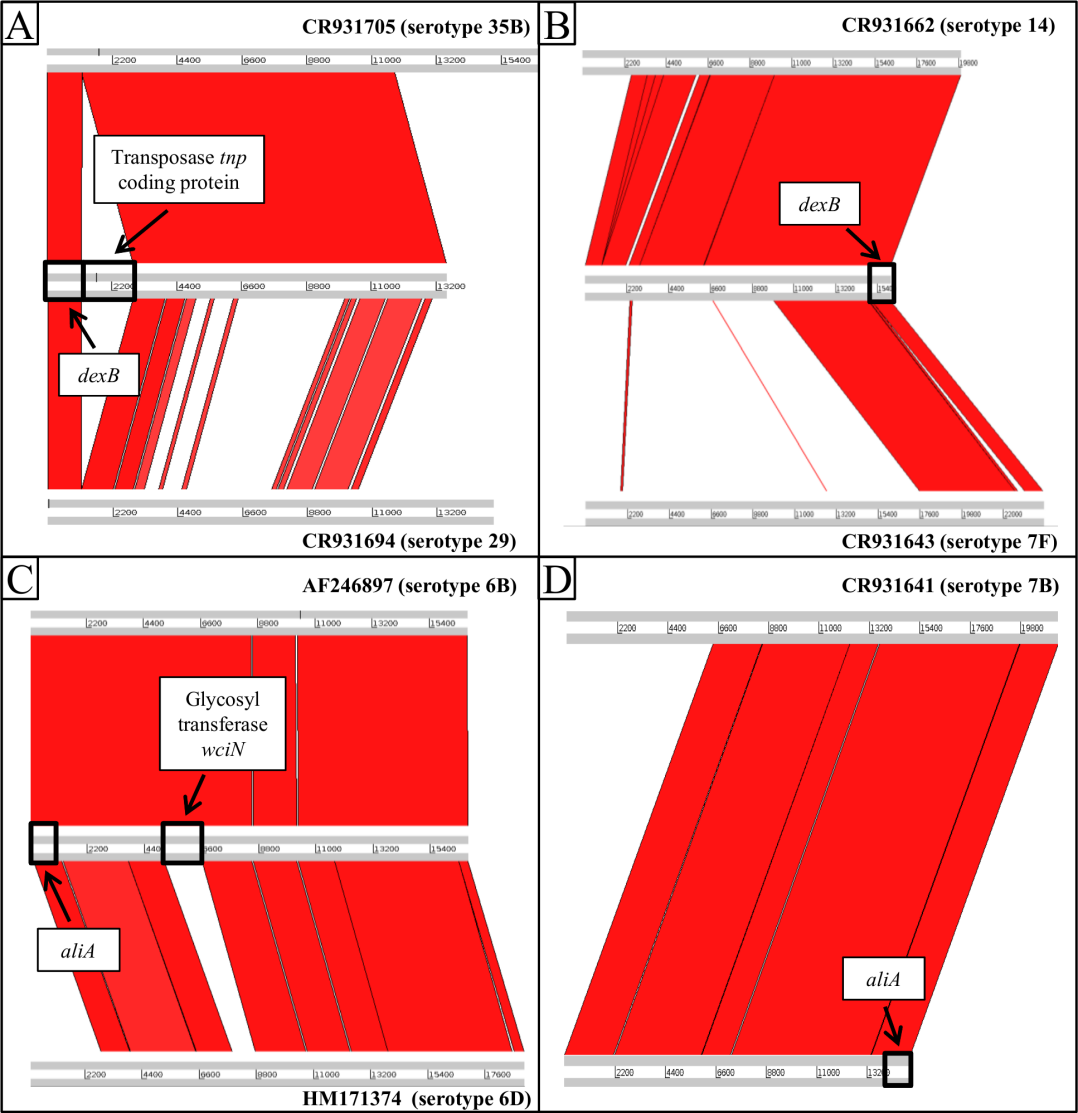
*cps* sequence alignments. Alignment of *cps* loci of serotype 29 isolate (A), serotype 7F isolate (B), serotype 6D isolate (C) and serotype 18B isolate (D) with reference *cps* sequence and best hit cps sequence according to WGS identification. Alignment was generated with Artemis Comparison Tool (http://www.sanger.ac.uk/science/tools/artemis-comparison-tool-act).

The *cps* locus sequence of misidentified isolates (serotypes 6D, 7F and 29) were aligned with the corresponding best hit reference sequence given by the in-house serotyping method and with the expected serotype sequence (Fig 2A–2C). No significant hit with 18B reference sequence was found for the misidentified serotype 18B isolate. Therefore, the *cps* locus was aligned with the best hit reference sequence (Fig 2D).

The *cps* locus alignment of our serotype 29 isolate resulted in fragmented hits with low identity compared with the serotype 29 reference sequence. The region 1174–2915 bp of our serotype 29 isolate sequence did not match with both serotype 29 and serotype 35B reference sequences and coded for a *tnp* transposase. It appeared that the *cps* locus of the serotype 29 isolate was located at the end of the corresponding contig and may be incomplete, resulting in a 1303 bp shorter sequence compared to the serotype 29 reference sequence. A very poor alignment was also obtained for our serotype 7F isolate *cps* locus sequence compared with the serotype 7F reference sequence, with less than 50% of the *cps* locus sequence correctly aligned. For the serotype 6D isolate, the major difference between the 2 alignments was the absence of a match with the serotype 6D reference sequence in the 5170–6608 bp region coding for the glycosyl transferase *wciN*.

PneumoCaT was also used for serotype attribution using the same set of WGS data (reads data). If a capsular typing variant analysis occurred, the serotype resulting from this analysis was retained for the serotype prediction. Sixty-one of 87 isolates (70%) were successively identified at the serotype level and the remaining isolates (30%) were misidentified. Considering only serotypes, 31 of 52 serotypes (59.5%) were identified at the serotype level and 19 of 52 (36.5%) were misidentified. Inconsistent results were obtained for serotypes 7F and 11A, representing 4% of the serotypes tested. Half of the misidentifications were due to an incorrect capsular typing variant analysis, resulting in this high proportion of misidentified isolates. One isolate (47A) could not be serotyped due to a too low number of reads due to contamination with *B*. *subtilis*, drastically decreasing the number of *S*. *pneumoniae* reads.

## Discussion

*S*. *pneumoniae* serotyping has become critical since the release of the different PCV for the monitoring of putative emergent NVT. Unfortunately, the gold standard Quellung method is expensive and laborious and can lead to interpretation errors. The implementation of a new and reliable serotyping method is needed, especially for surveillance programs such as the provincial surveillance held at the Laboratoire de santé publique du Québec.

In this study, 3 different molecular based serotyping methods (sequential multiplex PCR, sequetyping and WGS) were compared in order to evaluate their efficiency in serotype attribution for *S*. *pneumoniae* invasive isolates. This is the first comparison between these 3 methods on a common set of isolates.

PCR methods are very powerful, reliable and easy to perform. Multiplex PCR is an even more efficient technique since one single reaction allows the simultaneous detection of more than one gene and/or allele. The CDC sequential multiplex PCR method gave the expected results, with 27%, 29% and 20% identifications at the serotype, serogroup and subset level, respectively. This was also the method presenting the least misidentified isolates (1%). However, serotypes among a serogroup are inevitably revealed under the same signal in the current protocol due to their high level of genetic homogeneity. For example, primer pair 6A/6B/6C/6D in reaction 1 is simultaneously specific to four different serotypes. This is the most important limit for the efficiency of this method because no better results can be expected. Moreover, a significant number (23%) of serotypes were not detectable by this method, representing another limitation from a surveillance perspective. It also seems that small genetic variations in some isolates (serotype 35A) could determine the presence or absence of amplicon [30]. It is possible that the isolates tested were genetic variants of the CDC isolates of serotype 35A and that the primers 35A/35C/42 were unable to match these isolates. This finding would mean that the method efficiency could vary from one geographic region to another depending on the genetic distance with the isolates used for primer design. Another important aspect is the specificity of the method for *S*. *pneumoniae*. Indeed, it is not uncommon to confuse *S*. *pneumoniae* with other *Streptococcus spp*. due to their high degree of similarity, especially *S*. *pseudopneumoniae* [31]. Here, the internal control (*cpsA*) allowed differentiation between *S*. *pneumoniae* and *S*. *pseudopneumoniae* or *S*. *mitis*. However, 2 serotypes (25F and 38) were also negative for *cpsA* amplification making this discrimination not fully reliable. Finally, non-specific amplifications occurred during the study, as specified by the CDC (https://www.cdc.gov/streplab/pcr.html). Although most of the non-specific products did not match with expected amplifications, some of them could lead to misidentification.

Sequetyping is not limited to the number of detectable serotypes as *cpsB* sequences of almost all serotypes are present in regularly updated public database. Nevertheless, *cpsB* is not amplifiable in all serotypes, making these serotypes not identifiable with this method. This was the case for serotypes 25F, 37, 38, 39 and 43 in our study. Sequences for serotypes 39 and 43 were predicted to be non-amplifiable by Leung *et al*. (2012) even though they were amplified in their study. However, they did not obtain any amplification for serotype 25F or 38, which is consistent with our results. Finally, serotype 37 *cpsB* sequence was predicted to be amplifiable but was not tested *in vitro* in their study.

We decided to use the local *cpsB* sequence database created by Jin *et al*. (2016) instead of the database used by Leung *et al*. (2012) because this database was more comprehensive and covered more serotypes. Overall, we obtained more identification at the serotype level and less misidentifications using the local *cpsB* database as compared to the GenBank database. Significant differences were obtained for serotypes 6B, 6C, 19F and 23F where results between isolates of the same serotype were concordant with the *cpsB* database but not with GenBank database. Only well characterized sequences with full-length *cpsB* were chosen for this database and can explain these results. Indeed, slight variations in the *cpsB* sequence could have a major influence on serotype attribution when the GenBank database is used due to a lot of *cpsB* sequences presenting nucleotide variations not representative of the serotype. In contrast, the use of a local *cpsB* database with few but representative sequences avoided these mistakes. Apart from serotypes 12F, 17A, 18C, 24F, 29 and 35A, no equivalent data are available in Leung *et al*., (2012) for the other misidentified serotypes we observed in this study. For serotype, serogroup, and subset levels identification, our results are generally the same as the ones obtained by Leung *et al*., (2012). However, Comparisons are not always possible since 38 of our serotypes are missing in the Leung *et al*., (2012) study. Most of misidentified serotypes had some nucleotides of difference (from 1 to 59) with the best hit sequence, usually of the same serogroup or genogroup [28]. This is caused by intra-serotype variation [29] in the *cps* regulatory region and can lead to identification in the wrong serogroup. This issue has already been observed by Leung *et al*., (2012) with one 19F isolate identified as a serotype 1. Furthermore, some serotypes may have identical *cpsB* sequences as it is the case with some 6A and 6B isolates [32]. Moreover, for our serotypes 17A and 29 isolates, no significant hits were obtained with serotype 17A and 29 *cpsB* sequences, respectively. *S*. *pneumoniae* genome diversity may be high between geographically distant locations, leading to divergence between serotype 17A and 29 *cpsB* sequences present in the databases and sequences obtained in this study. However, this appears to be very unlikely [33]. Our evaluation of the sequetyping approach has demonstrated that this serotyping method is not always able to correctly identify serotype probably due to short DNA sub region of a large locus used in this analysis. Of the 6 other non-*S*. *pneumoniae* isolates tested, only one *S*. *pseudopneumoniae* led to a *cpsB* amplicon. This was not expected as it has been reported that *S*. *pseudopneumoniae cps* locus is not complete compared to *S*. *pneumoniae* and does not contain *cpsB* [34]. However, the low identity of the best HSP (96%) could help to discriminate this isolate. A recent method based on sequetyping including a second analysis step for homologous strains allowed to obtain more accurate results for these strains [35]. Such protocol could putatively help to obtain better results and make sequetyping more attractive.

Two different approaches were used for serotype identification using WGS method. Our in-house workflow consisted in assembling contigs from sequencing data and to BLAST them with a *cps* loci sequence database. Eighty-two percent of serotypes were identified at the serotype or serogroup level, demonstrating the efficiency of this strategy. Regarding unresolved serotypes (7A/7F, 9A/9V, 11A/11D, 12A/12F/44/46, 18B/18C, 22A/22F, 25A/25F, 32A/32F, 33A/33F and 35A/35C/42), these were all identified as another serotype belonging to the same genogroup as defined by Kapatai *et al*., 2016. More sensitive genetic analysis methods would be required to make a more accurate identification such as the capsular variant analysis integrated in PneumoCaT (see below).

Interestingly, serotype 22F isolates matched serotypes 22F/22A but with two separate HSPs. This unexpected BLAST result is caused by the high divergence of two genes (*wcwA* and *wcwC*) in the *cps* locus of those isolates compared to their orthologous sequences in serotype 22F. Similar finding were reported for isolate 1772-40b (GenBank accession HE651318; Salter *et al*., 2012), a 22F serotype which matches perfectly with our 22F isolates [36].

A serotype 29 isolate was misidentified with WGS and identified as serotype 35B. Serotype 35B and 29 are known to be genetically related, leading to cross-reactivity in antisera reactions [37]. However, no significant hit with serotype 29 was found in BLAST results, meaning that no relevant alignment could be made. These results were in agreement with sequetyping results obtained for serotype 29 isolates. Alignment with serotype 29 reference sequence (isolate 34373, Bentley *et al*., 2006) showed low identity although the serotype was confirmed by Quellung. Transposase coding region (*tnp*) was found downstream the *dexB* gene in the serotype 29 isolate. According to Bratcher *et al*., 2011, those regions may contribute to the vertical exchange of the *cps* locus between pneumococcal isolates and hence to their molecular evolution and adaptation, which could explain the low identity with serotype 29 reference sequence [38]. Serotypes 6D and 6B belong to the same genogroup. However, the glycosyl-transferase *wciN* is present in the 6B *cps* locus and not in the 6D *cps* locus, distinguishing those [39]. This gene was present in the studied serotype 6D isolate, which explains the misidentification with serotype 6B. It has been suggested that serotype 6D could have emerged from recombination between serotypes 6B and 6C but Song *et al*. (2011) highlighted the implausibility of this event because of a high genetic distance between these serotypes. Therefore, this gene acquisition was probably due to homologous recombination events or horizontal genetic transfers. The misidentification of serotype 7F (SC0218) isolate with serotype 14 and serotype 18B (SC0049) with 7B were surprising as these 2 serotypes belong to different genetic clusters [26]. These misidentifications cannot be attributed to sequencing quality as these 2 samples showed very good assembly’s length, coverage and N50 values (see S3 Table). Moreover, no cross-reactivity reactions are known between those serotypes (Statens Serum Institut, 2013, Key to pneumococcal factor antiseria. Accessed October 18, 2017. Available from http://www.ssidiagnostica.com/-/media/Admin/Diagnostica-Downloads/Downloads-UK/Brochures/BrochurePneumococcal-factor-antisera-key-18058.ashx).

PneumoCaT is the second approach we used for WGS serotyping and totally integrates a capsular variant analysis step in its workflow. Single Nucleotide Polymorphisms (SNPs) analysis, allelic variations or presence/absence of genes are analyzed when more than one locus is matched or if the match corresponds to a defined genogroup [26]. Although the first step gave results similar to the results obtained with the assembly-based approach, the variant-based step identifications did not match with Quellung identification for half of the serotypes tested. However, concordance between PneumoCaT and Quellung identifications were found for 8 serotypes (7A, 9V, 12A, 12F, 15C, 22A, 22F and 33F) which were only identified at the serogroup level or subset with the assembly-based approach. *S*. *pneumoniae* is in constant evolution, resulting in the apparition of genetic variants [40,41]. Recent publications have highlighted genetic variations present in the *cps* genes of certain serotypes leading to potential regional allelic differences [42–46]. CPS allelic variants in our isolates may explain the misidentifications occurring at the capsular typing variant analysis step. The possibility of local genetic variations between serotype-specific genes may require regular updating of the PneumoCaT databases to prevent these misidentifications.

Another automated serotyping pipeline for *S*. *pneumoniae*, SeroBA, was recently developed and used a hybrid assembly and mapping approach [47]. Although the authors proved that it was faster and less computational-intensive than PneumoCaT, serotype identification efficiency was equivalent between the two pipelines. Further comparative studies are required to fully evaluate the pipeline.

The aim of this study was to evaluate 3 DNA-based *S*. *pneumoniae* serotyping methods which could eventually replace the current Quellung gold standard method. Above all, none of the methods tested showed enough efficiency to be able to completely replace the Quellung method in surveillance programs. Indeed, identifications at the serogroup level were obtained with all of them but more particularly with sequential multiplex PCR. Though WGS produces reliable serotyping results, currently this method is still costly and time consuming. Nevertheless, with the automation of bioinformatic pipelines and the constant drop of reagent costs, this method could become very attractive for monitoring invasive *S*. *pneumoniae serotypes*. Moreover, the great amount of information generated with WGS can be easily valued with, for example, the analysis of molecular evolution of the isolates, the identification of putative vaccine targets in addition to the detection of antibiotic resistance and virulence genes. The sequential multiplex PCR and sequetyping strategy unlike WGS have specifically been developed to improve the serotyping response time and to reduce the associated costs. PCR has the inconvenience of requiring an adaptation to the local epidemiology of circulating serotypes. Simply changing the sequential order of the reaction may be sufficient but more often reviewing the combination of primers in the reaction mixture is needed.

In this study, we have demonstrated that WGS was the most reliable method among the 3 methods tested for serotyping of *S*. *pneumoniae*. However, serotype validation with Quellung is still required as some serotypes cannot be clearly distinguished with the *cps* sequences. Sequential multiplex PCR and sequetyping have the advantage to be cheaper than WGS and could also serve as a guide for Quellung method. But these methods have drawbacks making them less attractive. It is important to note that rare untypeable isolates, due to their lack of capsular polysaccharide, may generate a positive result with DNA based method [48]. In such cases, the final serotype identification would be in disagreement with the Quellung reaction which would produce a negative result. Conversely, the sequetyping or multiplex PCR approach may be used when the capsular swelling of the Quellung reaction is difficult to observe through microscopic examination. Finally, a total replacement of the Quellung reaction by a molecular method seems not possible yet. Nevertheless, WGS appears to be a very promising tool and could replace the Quellung method in the near future with its extensive use and the development of databases.

## Supporting information

S1 Table *S*. *pneumoniae* isolates and serotypes included in this study. (XLSX)

S2 Table Serotypes and identification level determined using the multiplex PCR and sequetyping methods. (XLSX)

S3 Table WGS and assembly quality metrics. (XLSX)

S4 Table Serotypes and identification level determined with WGS methods. For PneumoCaT, the serotype chosen after the capsule variant analysis step is represented in bold.
(XLSX)

S5 Table Impact of N50 and coverage on identification quality. This test was realized using a two-way Wilcoxon-Mann-Whitney test with an alpha value set to 0.05.
(XLSX)
